# The basics of commonly used molecular techniques for diagnosis, and application of molecular testing in cytology

**DOI:** 10.1002/dc.25067

**Published:** 2022-11-08

**Authors:** Scott A. Turner, Rand Abou Shaar, Zhongbo Yang

**Affiliations:** ^1^ Department of Pathology Virginia Commonwealth University Richmond Virginia USA; ^2^ Department of Pathology Roswell Park Comprehensive Cancer Center Buffalo New York USA

**Keywords:** molecular diagnostics, next‐generation sequencing, quantitative PCR, Sanger sequencing

## Abstract

Molecular diagnostics has expanded to become the standard of care for a variety of solid tumor types. With limited diagnostic material, it is often desirable to use cytological preparations to provide rapid and accurate molecular results. This review covers important pre‐analytic considerations and limitations, and a description of common techniques that the modern cytopathologist should understand when ordering and interpreting molecular tests in practice.

## 1 INTRODUCTION

The rapid advance of molecular techniques, especially next‐generation sequencing (NGS), makes molecular testing feasible in daily practice.^1^, ^2^, ^3^ Molecular genetic/genomic testing plays a vital role in diagnosis, prognosis, and prediction to therapy by testing various biomarkers in a number of diseases and has become an essential part of personalized medicine.

Cytopathology has been a tremendous diagnostic tool for examining morphology at the cellular level. The cytology samples are obtained by minimally invasive procedures that are usually well tolerated by patients, especially patients with comorbidities. Through utilization of cytology samples, pathologists can make a morphologic diagnosis that is associated with risk of malignancy to help with patient management. However, cytopathology has its limitations, the most common being limited diagnostic material in cytology samples. Molecular testing, in these situations, can really help refine morphologic diagnosis by further risk stratification, as well as providing additional prognostic and predictive information to help select the right patients for personalized treatment.^1^, ^4^ This is especially important for patients who are in the advanced stages of their diseases and cannot tolerate more invasive procedures, making cytology samples the only available material for molecular testing.

Various preparations can be made from cytology samples, including direct smears, liquid‐based cytology (LBC), and cell blocks. Different fixatives are used in these preparations. In general, non‐formalin fixed cytology materials are more suitable for molecular testing than formalin‐fixed tissue or cell blocks, mainly because these samples provide well‐preserved high‐quality nucleic acids that are easily extractable and stable.^4^, ^5^, ^6^ Even though the cytology samples are better material for molecular testing; they have been under‐utilized and less known to many pathologists and treating clinicians.^7^ Currently, most molecular tests are performed in formalin‐fixed paraffin‐embedded (FFPE) samples, mainly because these tests are validated on FFPE samples.^4^, ^5^, ^6^ However, many studies have shown that cytology preparations such as smears and LBC have at least comparable, if not better, performance in molecular testing than FFPE samples.^7^, ^8^ Therefore, cytology samples could be an excellent resource for molecular testing if samples are properly collected and molecular tests are carefully validated. In addition, rapid on‐site evaluation (ROSE) performed by cytopathologists can help ensure adequate samples are obtained for downstream molecular testing.

While most molecular testing is performed on FFPE tissue samples there are several well‐established molecular diagnostic assays, commercial and lab‐developed tests (LDT) that can be performed with cytology samples.^4^ For instance, molecular testing in cytologically indeterminate thyroid nodules can help further risk‐stratify these nodules. Afirma (Veracyte, Inc.) and Thyroseq (University of Pittsburgh Medical Center, Pittsburgh, PA, and CBLPath, Inc.) assays are widely used commercially available molecular tests for this indication.^9^, ^10^, ^11^ NGS‐based molecular testing has been applied in cytology samples obtained by endobronchial ultrasound‐guide fine needle aspiration (EBUS‐FNA) to molecular profile non‐small cell lung cancer (NSCLC) to detect potentially actionable mutations in select patients.^10^, ^12^, ^13^, ^14^, ^15^, ^16^, ^17^, ^18^ The same samples can also be utilized to perform PD‐L1 immunocytochemistry to select and predict immunotherapy treatment response.^19^ Molecular testing can also be used in cytology samples obtained by endoscopic retrograde cholangiopancreatography (ERCP) to help make more definitive diagnoses of lesions in the pancreaticobiliary tract.^5^, ^20^, ^21^

As molecular testing has become essential for patient care and is often requested to be performed using cytology samples, pathologists must understand the basics of molecular diagnostic methodology, indications for molecular testing, and how to best utilize various cytological samples for molecular testing. As with all laboratory tests, good starting material is crucial for accurate test results. This review will focus on the pre‐analytical factors that may influence the reliability of molecular testing. Additionally, this review will summarize common molecular techniques utilized in molecular testing of cytology specimens in daily practice.

## 2 PRE‐ANALYTICAL CONSIDERATIONS

### 2.1 Slide preparation

There are more pre‐analytical variables in cytology specimens compared to routine FFPE tissue samples. There are different fixatives and staining methods used in cytology slide preparations. The common cytology specimens consist of direct smears, LBC, and cell blocks.^4^, ^5^, ^6^, ^22^, ^23^ The updated molecular testing guideline for the selection of lung cancer patients for treatment with targeted tyrosine kinase inhibitors from the College of American Pathologists (CAP), the International Association for Molecular Pathology (AMP), and the Association for Molecular Pathology (AMP) recommend that any cytology sample, with adequate cellularity and preservation, may be used for molecular testing (Table 1).^24^

**TABLE 1:** Advantages and disadvantages to cytology sample types used for molecular diagnostics

Preparations	Advantages	Disadvantages
Direct smears	High‐quality nucleic acids Allow for ROSE evaluation	Need additional validation Slide sacrificing Delay due to coverslip removal
LBC	Aspirated material rapidly collected and preserved Material maximized	ROSE not available Variable quality of nucleic acids depending on fixatives
CB	No need for additional validation Preservation of diagnostic material Multiple sections	Poor DNA quality May be paucicellular ROSE not available
Supernatant	High yield and quality of nucleic acids Preservation of diagnostic material Additional source	ROSE not available No morphologic evaluation

Abbreviations: CB, cell block; LBC, liquid‐based cytology; ROSE, rapid on‐site evaluation.

Direct smears are typically created during fine needle aspiration (FNA) procedures. Different types of glass slides can be used while producing these smears. Studies show that fully frosted slides keep the highest cell retention and minimal cell loss during fixation, compared to the positively charged slides and non‐frosted slides. However, dislodging tumor cells from fully frosted slides can be challenging. Therefore, fully frosted slides are less frequently used for nucleic acid extraction.^4^, ^25^ Direct smears typically are enriched for tumor cells with intact whole nuclei rather than fragments of nuclei on the cell block or FFPE histology slides. One cellular FNA slide can contain up to 1,000,000 tumor cells. The minimum number of cells in the current NGS tests typically require 1000–5000 tumor cells, with a minimum tumor percentage of 20%.^6^, ^26^, ^27^, ^28^ The cells can be extracted from the slides using scraping or cell lifting with Pinpoint solution. Direct scraping of archival slides has a higher nucleic acid yield than cell lifting.^4^, ^25^ Microdissection of whole cells on the smears will help with tumor enrichment. Direct smears can be both alcohol‐fixed or air‐dried and they are both suitable for isolation of high quality nucleic acids.^5^, ^6^, ^22^ Nucleic acids are better preserved in alcohol than in formalin. Many studies have shown improved nucleic acid quality and NGS performance with smears compared to cell blocks.^4^, ^5^, ^6^, ^22^ Direct smears are commonly stained with Papanicolaou or Diff Quik staining methods. It has been shown that using direct smears with both staining methods, nucleic acids can be successfully isolated for various molecular tests. This is also true of archived smears. One of the limitations of using direct smears for molecular testing is the potential medicolegal issue of sacrificing diagnostic material as the smears are not reproducible. In addition, most molecular assays are validated using formalin‐fixed tissues, so a new sample type, like direct smears, may require a separate validation to satisfy CLIA/CAP validation guidelines. CAP also recommends preserving the diagnostic material on smears; however, in cases where the diagnostic smears must be harvested for indicated ancillary testing, photographs of diagnostic material or digitization of the slides is acceptable replacements for the original diagnostic material.^6^, ^29^

Liquid‐based cytology is another common cytology specimen. The samples are fixed with CytoLyt (Hologic) or CytoRich Red (Fisher Scientific, UK), and the slides are usually stained with Papanicolaou staining. LBC slides have been shown to have minimal differences in adequacy and in mutation detection rate compared to direct smears.^6^, ^23^ The molecular analysis can be performed by either scraping off the cells from the monolayer slide or using the suspended sample in the fixative solution.^6^, ^30^ Microdissection is possible but is more challenging on the slides compared to the direct smears due to the constricted area of cell deposition on the monolayer slide.^6^, ^27^

Cell blocks have been used more frequently than other non‐formalin fixed cytology specimens for molecular testing. These cell blocks can generate multiple sections, allowing for diagnostics slides to be retained while providing material for molecular testing. Additionally, the similarity of these cell blocks to traditional histology blocks means that a molecular assay previously validated for using FFPE samples will also be validated for using cell blocks.^4^, ^6^, ^7^, ^8^, ^9^, ^10^, ^11^, ^12^, ^13^, ^14^, ^15^, ^16^, ^17^, ^18^, ^19^, ^20^, ^21^, ^22^ However, cell blocks have limitations similar to traditional FFPE samples. Insufficient cellularity of cell blocks is the most common problem encountered in molecular testing.^22^ In addition, the quality of nucleic acids extracted from the cell block material is not as high as from non‐formalin fixed cytology specimens.^30^ If tumor cellularity meets the minimal level of detection of the assay and nucleic acid amount meets minimal test specifications, paraffin scrolls can be cut from the block and placed directly into a microcentrifuge tube for nucleic acid extraction. Otherwise, unstained sections can be cut and nucleic acids can be extracted by cell lifting or scraping from the unstained slides.^6^

Supernatant fluids obtained after cell pelleting and centrifugation during cytology specimen preparation can also be used for molecular testing. These samples are commonly discarded at the end of preparation. However, there are nucleic acid residues in these supernatant solutions that can be extracted for potential future use in molecular testing.^4^, ^6^, ^22^ A number of studies have exploited this possibility and found that DNA extracted from supernatant of FNA of various organs can be reliably utilized in NGS analysis and the results are comparable with the FNA‐tissue derive DNA.^22^, ^31^, ^32^, ^33^, ^34^, ^35^, ^36^

### 2.2 Sample quality

Several factors should be considered when submitting material for molecular testing, including, total cellularity, level of necrosis present, and the tumor content of the specimen.^6^, ^28^, ^37^, ^38^, ^39^ One of the greatest barriers to obtaining molecular results using cytology specimens is the low cellularity often obtained during sample preparations. While the cellularity required can vary greatly and depends on the molecular assay's nucleic acid requirements, it is important to submit specimens with the highest cellular content for molecular testing. Additional consideration should be given to selecting samples with limited (<20%) necrosis. Higher levels of necrosis can increase the amount of poor‐quality nucleic acids present in an extract and further inhibit optimal PCR amplification.^40^ The percentage of neoplastic cells in a sample required for accurate molecular testing depends on the molecular method's sensitivity. While there remains some variability in this estimate, the general rule of thumb is to provide a sample with a tumor content twice that of the limit of detection of the assay. However, for assays with higher sensitivity, like NGS, many laboratories will increase that cut‐off to account for possible tumor heterogeneity. Submitting a specimen with the highest tumor content available will decrease the concern of a false negative result.

### 2.3 Nucleic acid extraction

The first step in any molecular assay is proper isolation, purification, and extraction of nucleic acids (DNA and/or RNA) from a prepared specimen. A spectrum of samples can be used, including frozen, fresh, or fixed tissue, aspirate smears, and blood. Aspirate smears are the most commonly submitted cytology specimens and should be provided on fixed non‐cover‐slipped slides or from deparaffinizing 5–20‐micron glass slide sections obtained from the corresponding FFPE cell blocks. Specific care needs to be taken when fixed or decalcified samples are sent for molecular testing because many of these reagents can result in DNA damage. Fixatives and decalcifying agents, such as 10% buffered formalin for fixation and EDTA for decalcification should be used to avoid nucleic acid degradation.

In the modern molecular laboratory, numerous extraction methodologies have been successfully employed to obtain high quality nucleic acids. The methodologies range from historical chemical extractions; to phenol‐chloroform and proteinase K‐based methods^41^, ^42^ to more modern physical extraction methods using magnetic beads or column‐based purifications.^43^, ^44^ While these methodologies differ in principle, they all serve to remove contaminants such as proteins and lipids that can inhibit downstream amplification techniques central to most molecular assays. Method selection will largely depend on technology availability, the volume of testing, and the types of nucleic acids utilized for testing. Methods that purify only DNA are typically used to identify single nucleotide variants, insertions and deletions, and some copy number variants. While methods to extract RNA are typically employed to detect gene fusions or changes in gene expression. For more comprehensive methods, including many NGS panels, both RNA and DNA are required. Therefore, strategies to extract total nucleic acids will be selected to minimize the amount of tissue needed for analysis.

Upon extraction, the nucleic acid yield and quality are assessed through absorbance methods (e.g., NanoDrop™, ThermoFisher) or fluorescence methods (e.g., Quibit, ThermoFisher). Using a UV–vis spectrophotometer, absorbance at a wavelength at which nucleic acids absorb light most strongly (260 nm or A260) is taken, and nucleic acid quantity is calculated using the Beer–Lambert law, which predicts a linear change in absorbance with concentration. It is important to note that both DNA and RNA absorb light at A260; therefore, other nucleic acid contaminants may overestimate the actual yield. Sample purity can also be evaluated by determining absorbance ratios of nucleic acids (A260) with absorption by aromatic rings in amino acids (A280), and absorption by organic compounds and chaotropic salts (A230). The higher these ratios (A260/A280 and A260/A230), the greater the purity with the A260/A280 ratio of highly pure DNA ranging between 1.7–2.0 and RNA 1.8–2.3. Modern fluorescence methods rely on fluorescent dyes that selectively bind to the specific nucleic acid being measured (i.e., double‐stranded DNA, RNA, etc.). This selectivity allows for greater specificity and sensitivity over traditional absorbance methods, especially at lower nucleic acid concentrations. These dyes will emit an excitation wavelength that a fluorometer can measure. Nucleic acid yield can then be calculated by comparing the amount of fluorescence in the sample with that of a known standard curve. The accuracy of the final concentration is therefore dependent on the standard curve, making appropriate selection of the reference material imperative.

## 3 ANALYTICAL METHODS COMMONLY USED IN CYTOPATHOLOGY

### 3.1 Polymerase chain reaction

Polymerase chain reaction (PCR) is the process by which a target of DNA or cDNA created from RNA is amplified to create millions of copies of a specific genomic region. This process allows for the detection of a wide variety of genomic variants including, single nucleotide variants (SNVs), also referred to as somatic point mutations, deletions and insertions, copy number variants, and gene fusions depending on assay design. This technique can be leveraged to detect a single known target or multiple targets in a single reaction (multiplex PCR) either qualitatively by end‐point PCR or quantitatively by quantitative PCR (qPCR or real‐time PCR).

All PCR reactions require a double‐stranded DNA or a cDNA template, short target gene‐specific oligonucleotide primers, DNA polymerase, the four deoxyribonucleotide bases (dNTPs), buffer, KCl, and MgCl_2_. The template contains the desired region of nucleotides to be assayed and must be double‐stranded. For single‐stranded RNA to be amplified by PCR, it must first be converted to a double‐stranded cDNA molecule. This process, known as Reverse Transcriptase PCR (RT‐PCR), requires a reverse transcriptase (RT) enzyme, a targeting oligonucleotide, and the same buffers and reagents used in a PCR reaction. The targeting oligonucleotide may be either specific to the RNA target of interest or, more commonly, universally bind all RNA to create a complete cDNA library. These universal oligonucleotides are typically a series of random hexamers or target the poly‐A tract of mRNA. Once the oligonucleotide hybridizes with the RNA, the RT polymerase will read and add nucleotide bases (dNTPs) to the template RNA strand resulting in one double‐stranded cDNA molecule ready for use in downstream PCR reactions.

Along with the appropriate double‐stranded template, each PCR reaction must contain oligonucleotide primers that will flank the region or regions of interest. Additional design modifications to primers, including the addition of fluorophores, may be considered depending on the downstream application. A thermostable DNA polymerase facilitates the addition of nucleotide bases to extend the primers making a copy of the template. The added buffers maintain the pH of the reaction, KCl aids in proper primer hybridization, and the MgCl_2_ enhances polymerase activity.

**FIGURE 1:**
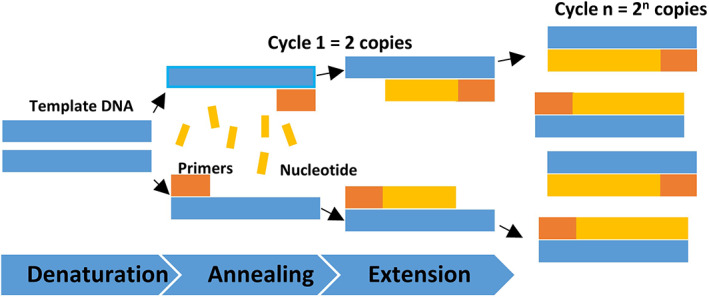
The three steps of polymerase chain reaction, denaturing, annealing, and extension during the first cycle and the exponential amplification with repeated cycling

The PCR reaction occurs inside an automated Thermal Cycler and consists of repeated cycles of temperature‐dependent denaturation, annealing, and DNA synthesis. In the denaturation step, the DNA double helix is broken by denaturing the bonds at high temperatures (>94°C), producing two single‐stranded DNA templates. The temperature is then lowered to allow for the annealing of oligonucleotide primers and probes to the DNA strand. Once annealed, the DNA polymerase can start catalyzing the elongation of the new DNA strand. This temperature cycling is repeated for a set number of times, dependent on the amount of amplification required for downstream application, with the result of 2 ^N^ copies of the targeted sequence, or amplicons, with N being the number of cycles performed (Figure 1).

The process by which these PCR products are detected and analyzed is dependent on the intended downstream application. This review will further discuss some commonly used applications including, qPCR, fragment analysis (Sanger Sequencing), and next‐generation sequencing (NGS).

### 3.2 Quantitative PCR

**FIGURE 2:**
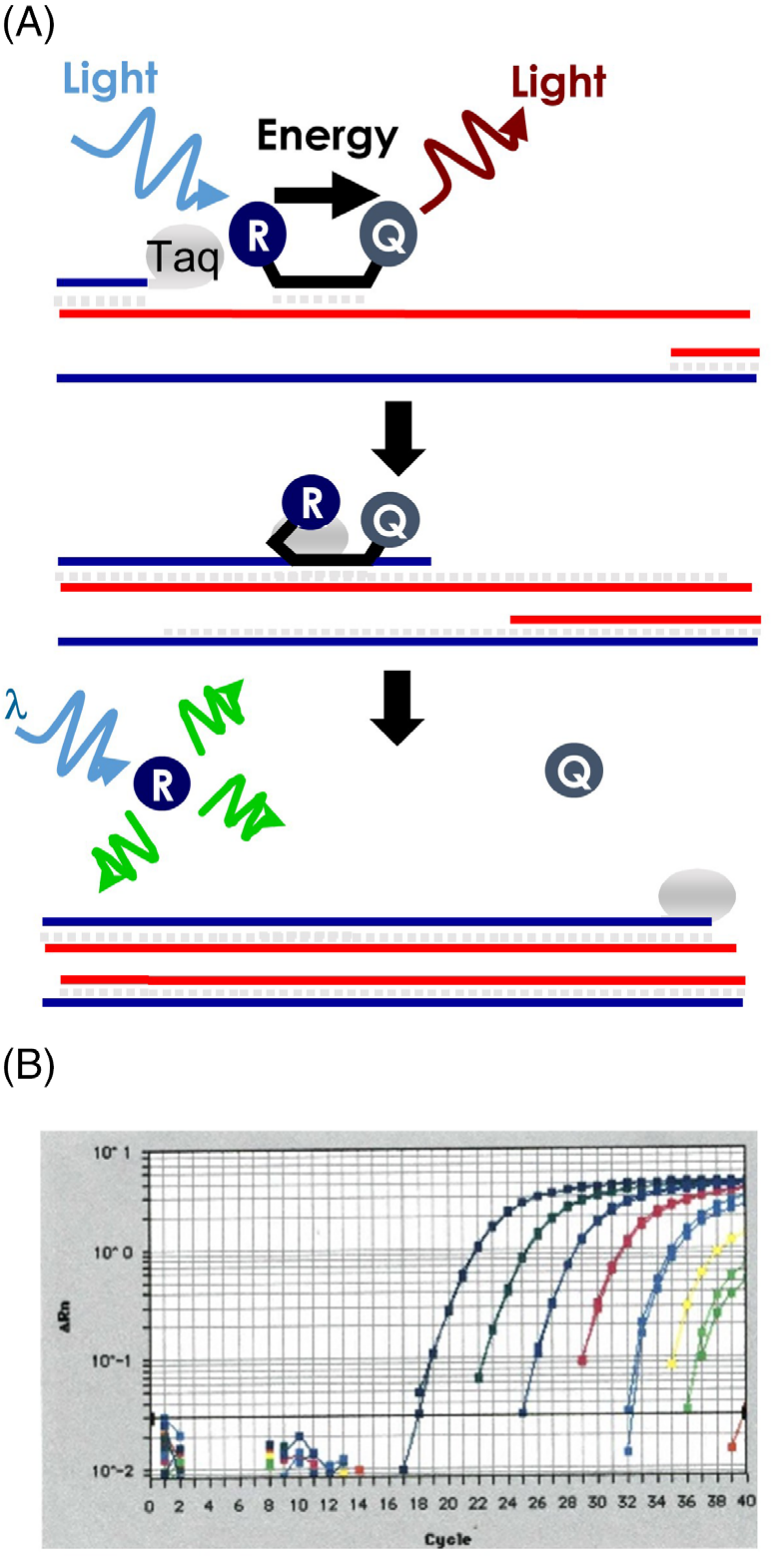
Taqman quantitative polymerase chain reaction (PCR). (A) Primers and probe anneal to complementary sequence. The oligonucleotide probe is labeled with a fluorophore (R) and a fluorescence quencher (Q). During the PCR extension step, the 5′ exonuclease activity of Taq polymerase digest the probe resulting in release of (R) from the (Q) allowing for the fluorescence detection of the reporter. (B) The real‐time detection of accumulating fluorescence at the end of each cycle allow for calculation of comparative template quantity

Some clinical applications, including identifying differential methylation, genotyping known single nucleotide variants, or quantifying gene fusions transcripts, require accurately determining the amount of a target sequence present in the source sample. To accomplish this, qPCR chemistries rely on the use of fluorescence‐labeled oligonucleotide probes added to a PCR reaction described previously. These probes are designed to hybridize within the amplified target region, providing increased specificity to the PCR reaction. When the fluorophore absorbs light energy at a particular wavelength, it simultaneously emits energy at a lower wavelength than a qPCR instrument can measure.^45^ Rather than detecting the amount of fluorescence at the end of the PCR reaction, qPCR will read the fluorescence at the end of every PCR cycle allowing for visualization of the exponential amplification of the target. To get around the problem of non‐specific fluorescence of unbound probes during cycling, these probes are designed to take advantage of the principles of fluorescence resonance energy transfer or FRET. The principal mechanism of FRET is the transfer of energy from one fluorophore to another when placed in close proximity.^46^ Probe designs vary based on the chemistry of the qPCR assay and the intended downstream application. However, one common qPCR method employed in the clinical laboratory utilizes the inherent 5′ → 3′ exonuclease activity of Taq DNA polymerase. The qPCR probe is end‐labeled with two fluorophores, a reporter and a quencher. When the probe is intact, these fluorophores are separated by approximately 20–25 base pairs resulting in energy transfer. The emission wavelength of the reporter excites the quencher, effectively silencing the reporter by removing any residual energy and preventing it from being detected. However, during the PCR reaction, as Taq polymerase acts to copy the template strand, it will displace and degrade the qPCR probe separating the reporter and the quencher. At the end of the PCR cycle, a reading will be obtained, all free reporters will emit the detected wavelength, and residual intact probes will remain silenced. The fluorescent signal will increase exponentially until one of the reagents is exhausted, at which point each cycle will no longer result in a doubling of the target (Figure 2).

To calculate the number of starting copies present, the original sample must be compared to a known standard. Amplification curves of samples and standards are compared using the number of cycles it takes to reach a predetermined fluorescence threshold (*C*_t_ or *C*_q_) value. This threshold must be within the exponential growth phase of all curves and will be dependent on the primers and probes used for the assay. A standard curve can be produced by determining the *C*_t_ value of the known standards. The *C*_t_ values of the known standards can then be used to determine a curve and by comparison to the relative quantity of the target in the starting material. Variations of this type of analysis make it possible to monitor things like tumor burden and minimal residual disease of a fusion using RNA transcripts.^47^ These types of analysis have also been adopted into semi and fully‐automated platforms like Biocartis Idylla™ that can go from FFPE sample to result in approximately 2 h.^48^, ^49^ This is covered in detail in other reviews.^50^, ^51^

### 3.3 Fragment analysis by capillary electrophoresis

The qualitative detection of PCR products is achieved through electrophoresis of amplified nucleic acid targets. In brief, in nucleic acid electrophoresis, an electrical current is applied to the negatively charged PCR product, which results in the migration of the product through a viscous medium allowing for size separation of the PCR products. These principles have been covered elsewhere in detail.^52^ In the clinical laboratory, agarose or polyacrylamide gel electrophoresis has largely been replaced by capillary electrophoresis. These instruments often contain multiple capillaries (8–96) filled with a viscous matrix that allows for accurate size separation that can be more easily scaled to the application and volumes required by the laboratory. Somatic applications include detection of pathogenic deletion and insertion,^53^ presence of pathogenic variation through restriction enzyme digestion,^54^ allele‐specific PCR,^55^ and Sanger sequencing.^56^

### 3.4 Principles of Sanger sequencing

**FIGURE 3:**
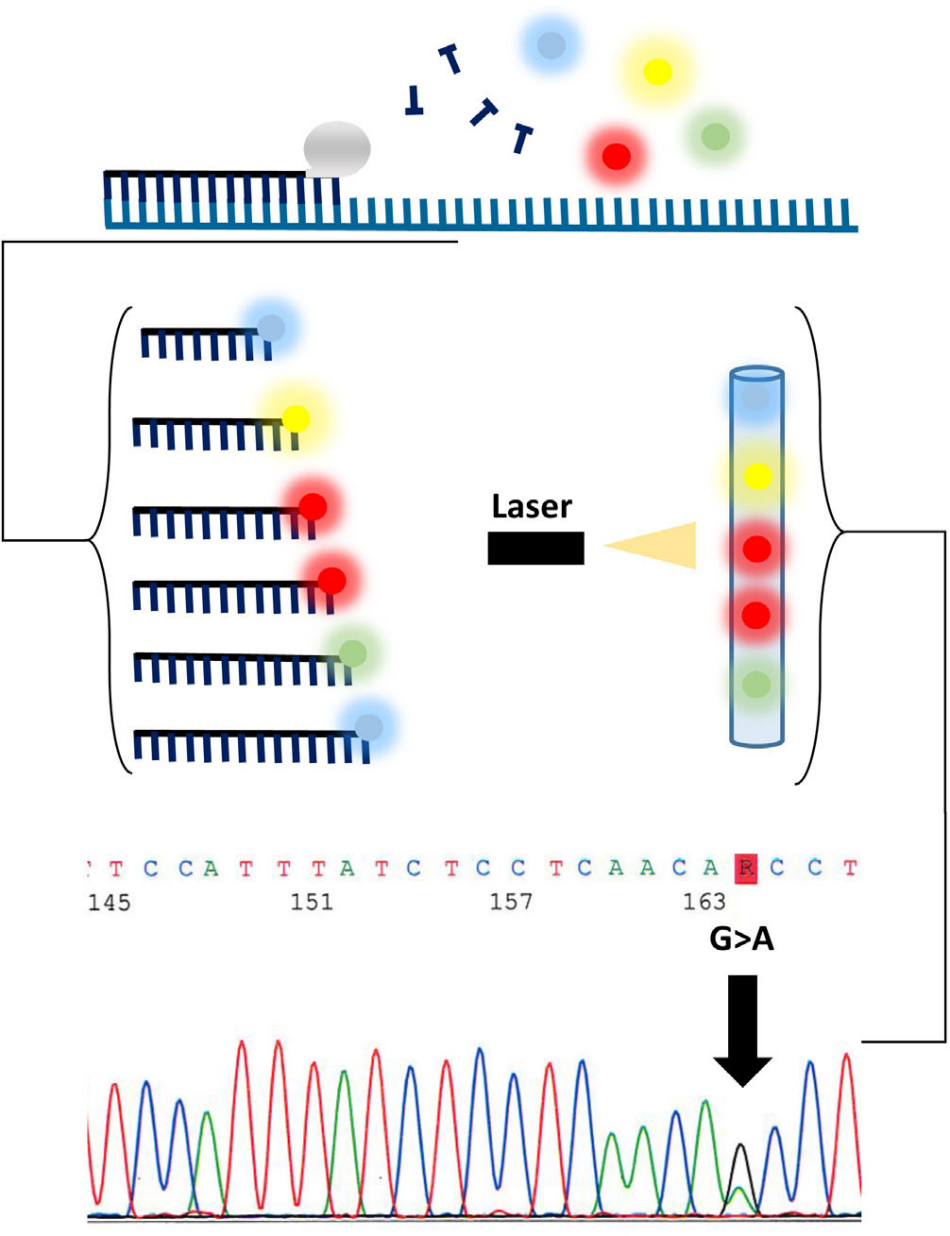
Sanger sequencing methodology. A complimentary sequencing oligonucleotide primer hybridizes with single‐stranded DNA. The strand extension results from addition of a mix of unlabeled deoxynucleosides tiphospates (dNTPs; dATP, dTTP, dCTP, and dGTP) and fluorescently labeled Dideoxynucleoside triphosphates (ddNTPs; ddATP, ddTTP, ddCTP, and ddGTP). When a ddNTP is incorporated in extending strand it result in extension termination. A fragment library is separated by size using capillary electrophoresis and fluorescent label is detected and converted into the sequencing signal

Sanger sequencing can analyze genomics regions of approximately 300–1000 base pairs in size. Following PCR amplification, the purified PCR products are subjected to a sequencing reaction that includes enzymes, buffers, a sequencing primer, and a mixture of dNTPs and fluorescently labeled dideoxynucleotides (ddNTPs). As the enzyme works to create a copy of the PCR template either a dNTP or a ddNTP is incorporated into the extending strand. When a ddNTP is added, it prohibits the subsequent addition of dNTPs resulting in the termination of the extending strand in a process termed dideoxy chain termination. This process results in the creation of a library of DNA fragments with a fragment terminating at every base of the sequence. This fragment library is then analyzed by capillary electrophoresis and the fluorophore tagged ddNTP in each fragment is identified. The final genomic sequence is determined by converting the identified fluorophore to the known ddNTP carrying the fluorophore. This process is simplified by using various software tools designed to assemble and compare these sequences to a reference sequence. Variation is identified when either multiple peaks (or bases) are present at the same location or when the sequence determined is different from the reference sequencing (Figure 3). Sanger sequencing has long been considered the gold standard for accurately detecting single nucleotide variants and deletions and insertions, as long as contained within the amplified PCR product. However, the applications for sanger sequencing of cytology specimens are limited by assay sensitivity with a tumor burden requirement of ~50% to avoid false negatives due to high tumor heterogeneity. However, many of the limitations of somatic sanger sequencing have been reduced through the wide adoption of advancing next‐generation sequencing methodologies.

### 3.5 Principles of next generation sequencing

Advancements in sequencing technology have allowed for the high throughput sequencing of numerous molecules of DNA/RNA simultaneously, referred to as next‐generation sequencing (NGS) or massive parallel sequencing (MPS). This has opened the door to faster and more cost‐effective methods for sequencing thousands of amplicons at a single time. With the advent of NGS, it is now possible to sequence genes targeted for diagnosis, treatment, and prognosis of specific cancers (cancer hotspot panels), the entire exome (whole exome sequencing, WES), or even the genome (whole genome sequencing, WGS).

**FIGURE 4:**
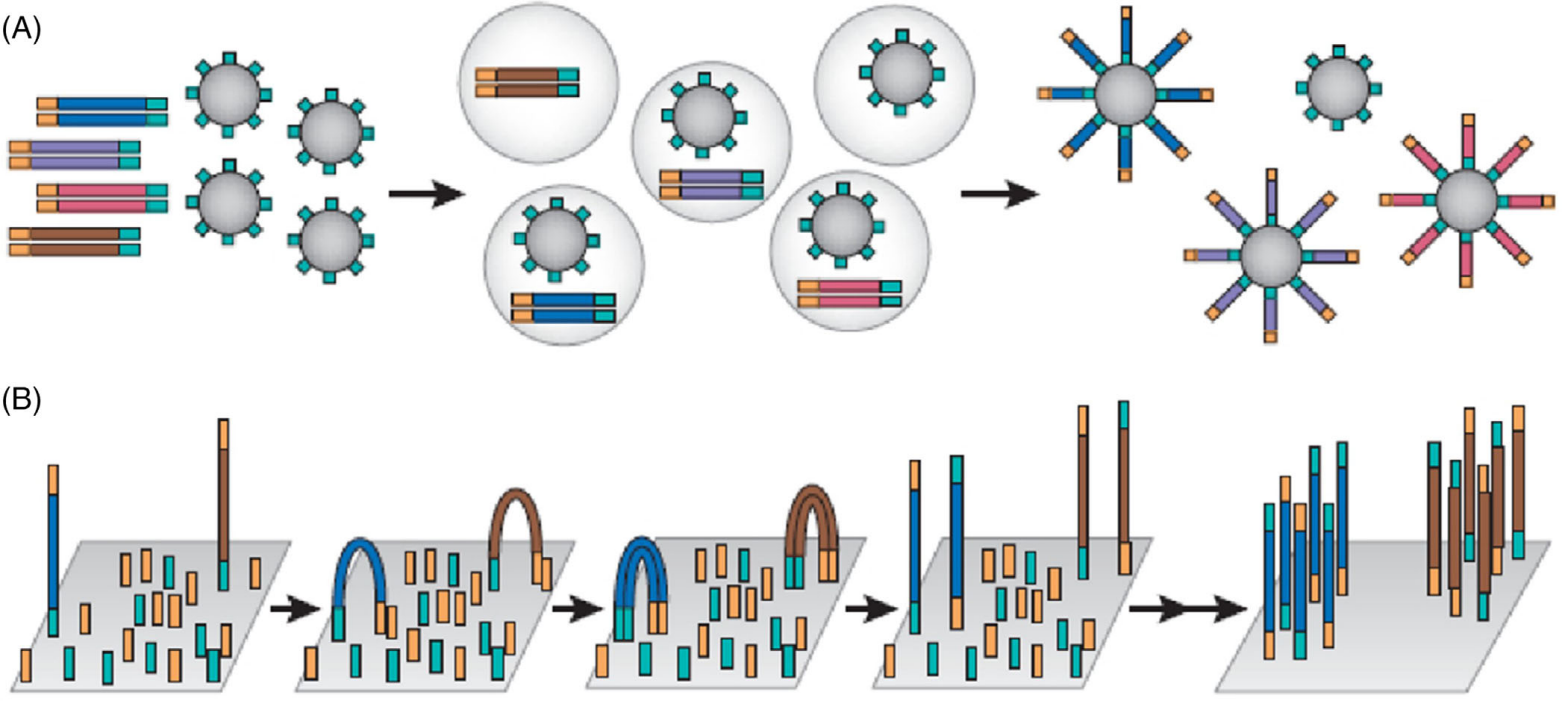
Clonal amplification methods in next generation sequencing. (A) Emulsion polymerase chain reaction (PCR) (IonTorrent); enriched adaptor labeled DNA products are combined with beads with complementary adaptor sequences. PCR amplification occurs in a water‐in‐oil emulsion with vesicles containing only one bead and one template. Amplicons are contained on the surface of the beads. Once complete the droplets are broken and residual beads contain enriched products. (B) Bridge PCR (Illumina); enriched adaptor labeled DNA products are injected across the glass surface of a flow cell bound with complementary adaptor sequences. The DNA product hybridized with the adaptor on the solid surface and undergoes bridge amplification. When PCR is complete a cluster of identical fragments will be ready for sequencing^61^

**FIGURE 5:**
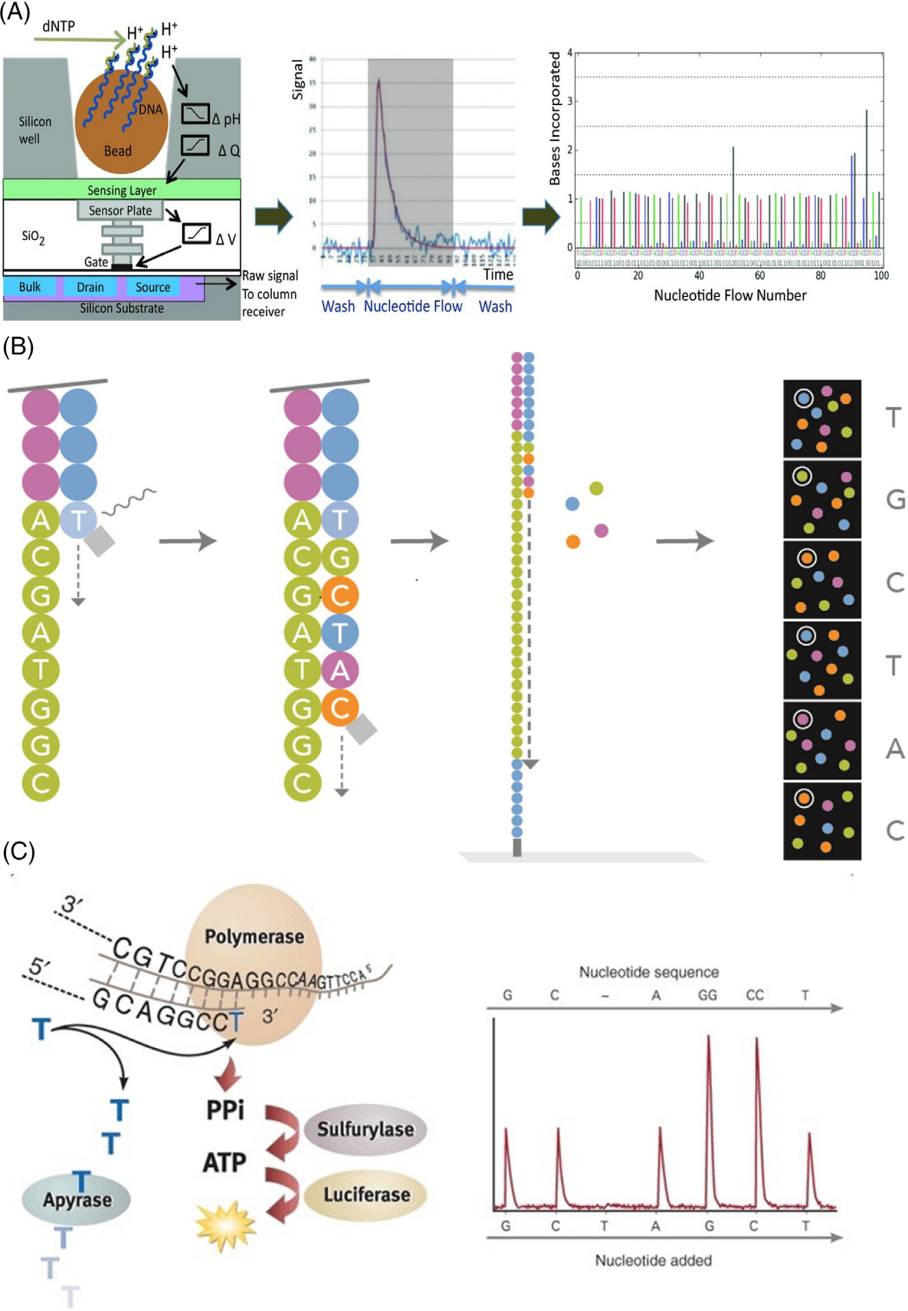
Sequence by synthesis (SBS) chemistries; (A) ion detection (IonTorrent) using a semiconductor pH device; beads templated with clonally amplified single‐stranded DNAs are deposited in the pH sensor wells. As specific dNTP (A, C, T, and G) flows into the well, a polymerase driven extension of the template strand incorporates the nucleotide, and a H^+^ ion is released. The H^+^ release is converted into an electric signal, which can be interpreted as sequencing signal. The process occurs simultaneously across 1000 s of wells on a sequencing chip. Used with permission of John Wiley and Sons^62^; permission conveyed through Copyright Clearance Center, Inc (B) Cyclic reversible termination (Illumina); after template enrichment via cluster generation, a mixture of DNA polymerase, primers, and dNTPs modified with a 3'‐O‐azidomethyl group and a base‐specific fluorophore are added to the flowcell service. The extending strand will incorporate one nucleotide and then blocks additional dNTPs incorporation. The slide is imaged using either two or four laser channels and the fluroescent signal identified indicates which base was incorporated into the sequencing. The extending strand can then be enzymatically prepared to incorporate the next available nucleotide completing a single cycle. The number of cycles correspond with the number of bases sequenced. Figure used courtesy of Illumina, Inc. (C) Pyrosequencing; after template enrichment as a new dNTP is added to the extending strand a pyrophosphate molecule (PPi) is created. The PPi molecular and added ATP sulfurylase converts adenosine 5′ phosphsulfate (APS) into ATP. ATP can then act as a cofactor to chemically convert luciferin to oxyluciferin which creates light. Each light burst can then be detected to indicate the sequence of the newly forming strand. Reprinted with permission from Springer Nature^63^

Most commercially available NGS platforms are capable of short‐read sequencing (~150–200 base pairs of sequence) through sequence by ligation (SBL) or sequence by synthesis (SBS) chemistries, which are reviewed in additional detail elsewhere.^57^ In short‐read sequencing, sequencing libraries are typically prepared by fragmenting and end‐treating genomic DNA or cDNA libraries to prevent overhangs and unwanted strand extension. Additional oligonucleotides, known as adaptors, are hybridized or ligated to DNA strands to allow for the unique identification of each molecule and its source. These adaptors allow for simultaneously sequencing specimens from many patients on a single chip or flow cell. These adaptors also add proprietary sequences required for hybridization to the sequencing substrate. Targets in hotspot sequencing panels common in somatic sequencing are enriched either through PCR‐based amplification or probe hybridization and purification methods. Enrichment of these targets increases sequencing coverage by reducing off‐target sequencing and maximizing usable sequencing output. Next, the enriched libraries are clonally amplified to produce a template library suitable for sequencing. This clonal amplification occurs on either a solid surface (e.g., Illumina) or in an emulsion (e.g., IonTorrent, RainDance) and the increased signal produced during the sequencing of each molecule increases the overall accuracy of the sequencing reaction (Figure 4). Upon completion of library preparation, target enrichment, and clonal amplification, the fragment library is ready for sequencing. Similar to Sanger sequencing, SBS chemistries determine base composition by detecting the signal that is emitted from newly incorporated nucleotides during the sequencing reaction. The signal capture technique differs depending on the sequencing platform. In SBS chemistries, the emitted signal consists of either a fluorophore (Illumina), a change in ionic concentration (Ion Torrent), or the detection of light (Pyrosequencing) (Figure 5).

While these short‐read NGS technologies are currently the most commonly performed sequencing assays in the clinical molecular laboratory, there are several limitations to this type of sequencing strategy. Short‐read sequencing generates reads that may not overlap one another, potentially decreasing target coverage. These sequencing chemistries have difficulty distinguishing complex long tandem repeats, as in centromeric regions and satellite arrays.^57^ This is particularly evident using Ion Torrent and pyrosequencing technologies when sequencing homopolymer regions. In addition, short‐read sequencing cannot determine on which allele the variant lies, or genetic phase. Another limitation is amplification bias, which occurs because GC or A‐ rich regions are amplified less efficiently. This limitation can introduce inaccuracies during the library preparation step after several cycles of amplification.

**FIGURE 6:**
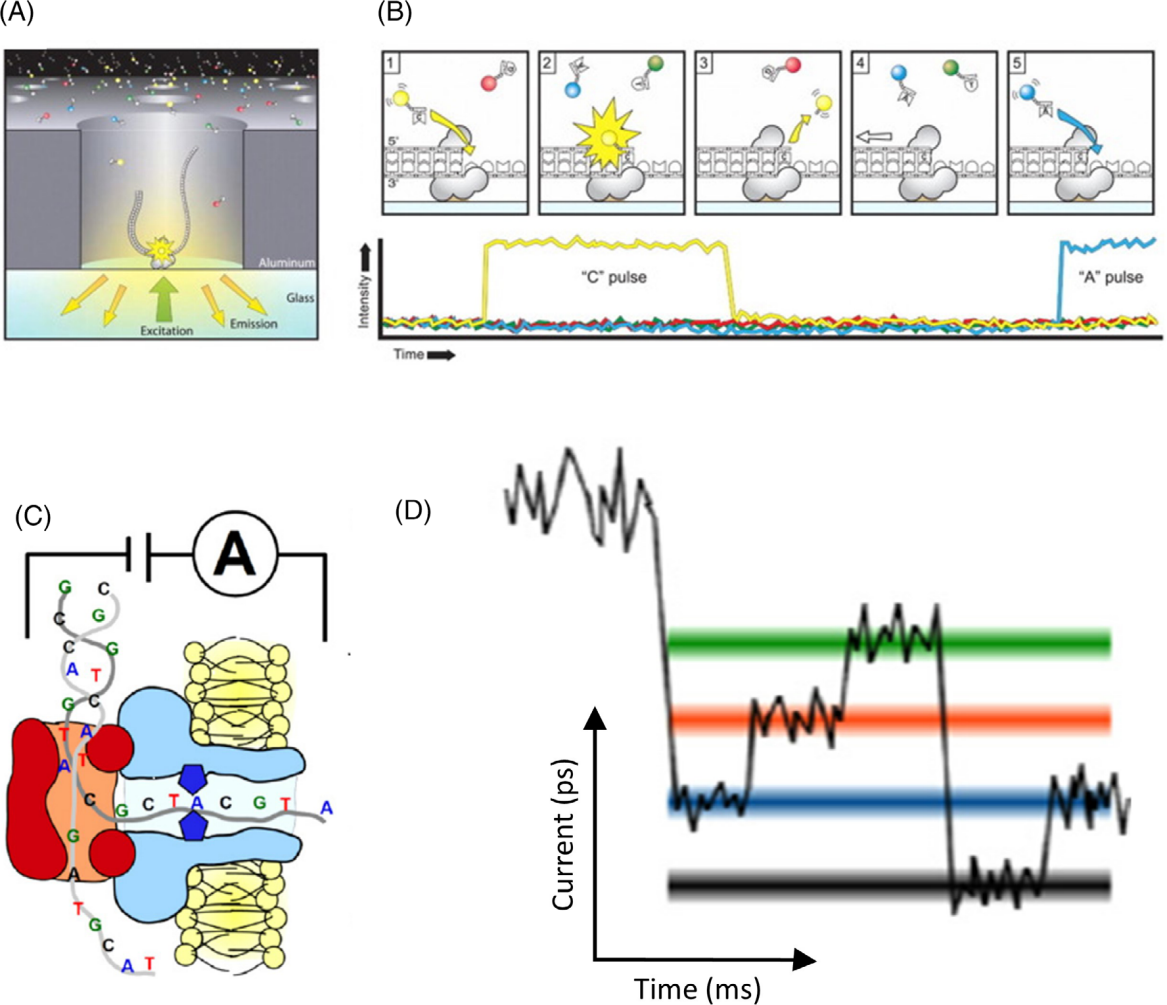
Long read sequencing chemistries from Pacific Bioscience and Oxford Nanopore; (A) A SMRTbell diffuses into a ZMW, and the adaptor binds to a polymerase immobilized at the bottom.^64^ (B) As a fluorescently labeled nucleotide is held in the detection volume by the polymerase, a light pulse is produced that identifies the base. The fluorescence output of the color corresponding to the incorporated base is elevated. A dye‐linker‐pyrophosphate product is cleaved from the nucleotide and diffuses out of the ZMW, ending the fluorescence pulse. Reprinted with permission from AAAS.^64^ C) Double‐stranded DNA is separated into single‐stranded DNA by a polymerase within the nanopore. The nanopore possesses a constriction inside the channel (dark blue diamond), which enables reading of the single‐stranded DNA sequence.^65^ (D) A simplified diagram showing the nanopore decoding method. The ionic current trace is altered by the DNA sequence translocating through the nanopore decoding the DNA. © IOP Publishing. Reproduced with permission.^65^ All rights reserved

Some of these limitations have been addressed by increasing the sequencing read length. These real‐time long‐read sequencing chemistries include the PacBio Single‐molecule real‐time (SMRT) sequencing and the Nanopore sequencing approach. In PacBio SMRT sequencing, two hairpin adaptors are ligated at the ends of the DNA template (called SMRTbell template) to allow for continuous circular sequencing. The SMRTbell template is then directed to the special zero‐mode waveguide (ZMW) wells, where sequencing is initiated. In the ZMW wells, fluorophore‐labeled nucleotides are added to the elongating DNA strand. The camera at the bottom of the well records the light emitted whenever a base is incorporated. Nanopore sequencer uses the native single‐stranded DNA fragment to detect the DNA composition directly. The DNA template for the nanopore consists of a double‐stranded lead adaptor that contains a specific sequence that helps guide the template into the charged protein pore. When the DNA strand passes through the pore, the voltage changes inside the pore and is recorded as DNA sequence (k‐mer). (Figure 6).

### 3.6 The NGS bioinformatics pipeline

**FIGURE 7:**
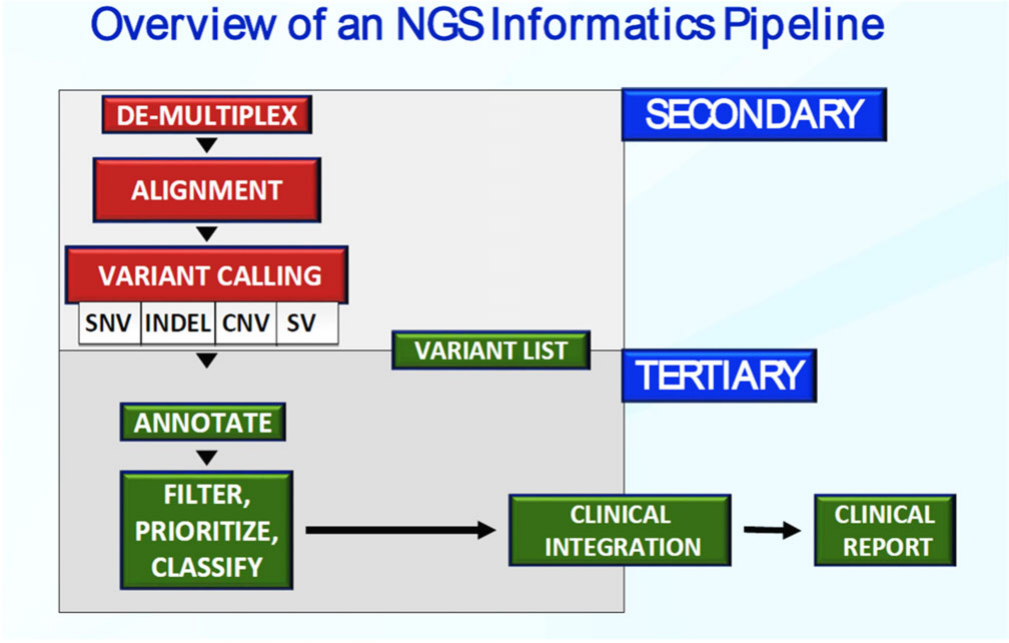
Simplified diagram of a bioinformatics workflow

Laboratories rely on several computational steps to make sense of data generated by NGS platforms. This bioinformatics process generally consists of three tiers; primary, secondary, and tertiary analysis (Figure 7). Following the wet lab process described earlier, samples are sequenced, and data regarding the base incorporated in the extending DNA fragment is generated. This primary analysis includes specifics about each base called per cycle and the quality of each call. The secondary analysis uses the raw sequence that was identified through primary analysis and aligns the sequences against a reference genome. Variant calling tools detect any variation from the reference in the samples sequenced. Several publically available algorithms exist, and many additional proprietary variant callers have been developed and incorporated into assay‐specific bioinformatics pipelines. Finally, the tertiary analysis consists of visualizing, filtering, and annotating the variation identified. Variant calling tools detect thousands of gene variants. However, many of those are clinically irrelevant because they are associated with common variations within a population, lie in regions of the genome for which clinical and function information is unavailable, or may represent pipeline‐specific sequencing errors or artifacts. Several parameters are used to filter these variants allowing for a more focused approach to manual variant review. Some examples of filtering parameters include; population polymorphisms, functional domain filters, read depths, and various sequencing quality metrics, including strand bias and call quality.^58^ The results of these well‐validated filtering steps are the removal of the great majority of irrelevant variation and the inclusion of a more manageable number of potentially pathogenic variants for manual review.

**FIGURE 8:**
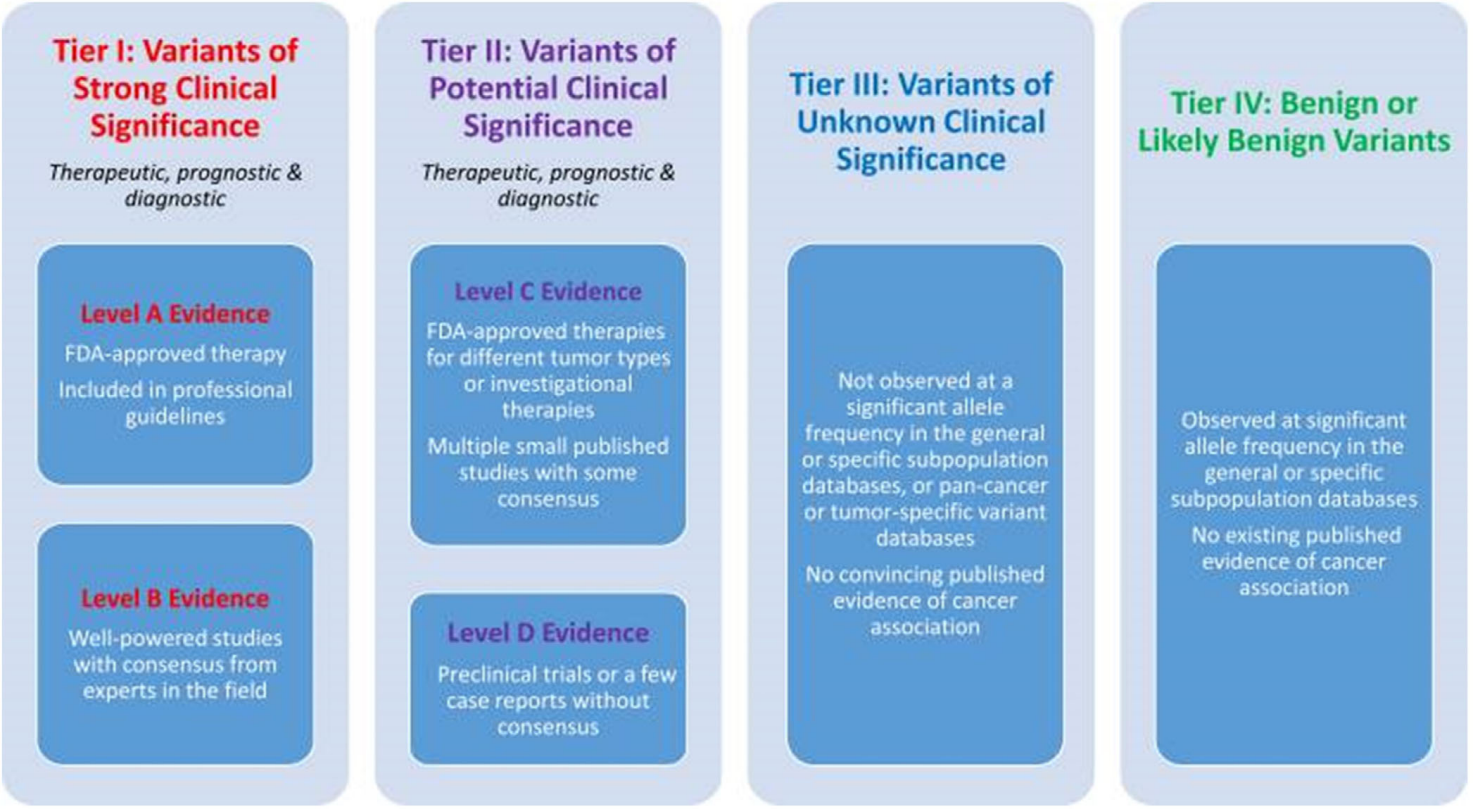
Evidence‐based clinical classification system for somatic variants. Reprinted with permission from Elsevier^59^

Variants are then classified on the evidence‐based strength of their prognostic, diagnostic, and therapeutic profile. In 2017, AMP/ASCO/CAP published guidelines to assist laboratories in classifying somatic variants to help standardize reporting of clinically relevant variants.^59^ In brief, variants with the strongest clinical significance in cancer (e.g., within national guidelines) for diagnosis, prognosis, or an FDA‐approved treatment are classified as tier 1, those with potential clinical significance in cancer (e.g., clinical trials, clinical or functional studies) are classified as tier 2, variants with unknown clinical significance (VUS) are classified as tier 3 and benign or likely being variants are classified as tier 4 (Figure 8).

### 3.7 Additional considerations for NGS

Historically, most standard NGS assays have a limit of detection of ~5% allele frequency. This limit of detection is determined by the number of reads (depth of coverage) for each base and the amount of sequencing error or background in the sequencing platform. To increase accuracy and error correction, a barcoding system has been implemented. These include unique molecular identifiers and unique dual index barcodes. They consist of short random DNA sequences that attach to the DNA library sequences, uniquely tagging each for later identification. Duplicates and false‐positive reads are parsed from rare variants as they go through the bioinformatics pipeline, referred to as error correction sequencing. This technology also allows for the digital quantification and tracking of clones and subclones for minimal residual disease measurement.^60^

## 4 CONCLUSIONS

The application of molecular testing, especially NGS technology, to cytology samples plays a significant role in diagnosis, prognosis, and prediction to the potential treatment of various diseases. Molecular cytopathology, therefore, is essential in the era of personalized medicine. Cytology samples typically contain better preserved nucleic acids compared with formalin‐fixed tissue samples. In addition, these samples can be easily obtained by minimally invasive procedures, and in many situations, they are the only available material for further testing. With proper validation, various cytology specimens can be utilized appropriately to help refine uncertain morphologic diagnoses, and provided critical prognostic and predictive information about treatment plans. The modern cytopathologist needs to be familiar with the basics of molecular testing in cytology samples, including pre‐analytical considerations, various common molecular techniques, and the clinical utility of these tests. With this knowledge, the cytopathologists will be better informed and more engaged in patient care in the era of precision medicine.

## CONFLICT OF INTEREST

The authors declare no conflict of interest.

## Data Availability

Data sharing is not applicable to this article as no new data were created or analyzed in this study.
